# Long-term safety and pulmonary function stabilization with nintedanib in systemic sclerosis-associated interstitial lung disease, 2020–2025: a real-world retrospective study from a reference centre

**DOI:** 10.1007/s00296-026-06173-6

**Published:** 2026-06-12

**Authors:** Stylianos Panopoulos, Vasiliki Poulia, Nikolaos Vlachogiannis, Vasiliki-Kalliopi Bournia, Maria G. Tektonidou, Petros P. Sfikakis

**Affiliations:** First Department of Propaedeutic and Internal Medicine and Joint Rheumatology Programme, Medical School, National and Kapodistrian University of Athens, Μedical School General Hospital of Athens ΄΄LAIKO΄΄, 17 Agiou Thoma street, 11527 Athens, Greece

**Keywords:** Systemic sclerosis, Interstitial lung disease, Immunosuppresives, Retrospective study

## Abstract

Randomized trials have demonstrated that nintedanib is beneficial for Systemic Sclerosis-associated interstitial lung disease (SSc-ILD) with an acceptable safety profile. We evaluated the long-term safety and efficacy of nintedanib in a real-world SSc-ILD cohort. Medical records of SSc patients receiving nintedanib for newly diagnosed fibrotic or progressive ILD between 2020 and 2025 in our center were retrospectively reviewed. Forced vital capacity (FVC%) and diffusing capacity for carbon monoxide (DLCO%), were recorded 12 months before and 12/24/36 months after nintedanib initiation and compared by Wilcoxon signed-rank test. Safety analyses included all treated patients; efficacy analyses excluded patients initiating immunosuppression simultaneously with nintedanib. Fifty-one patients (41 female; 34 diffuse SSc; median age and disease duration of 53 and 6 years, respectively) received nintedanib for a median of 33 months (range 4–60). Diarrhea was the most frequent adverse event leading to dosage reduction in 31% and permanent discontinuation in 14% of patients, respectively. No additional safety signals emerged in patients receiving mycophenolate (*n* = 18), tocilizumab (*n* = 14), rituximab (*n* = 1), mycophenolate plus tocilizumab (*n* = 5) or mycophenolate plus rituximab (*n* = 2). FVC% and DLCO%, that had declined significantly during the preceding year, remained stable after nintedanib initiation in 23/35 patients with median FVC changes of + 1% and − 3% and median DLCO% changes of -2% and − 4% after 24 and 36 months, respectively), including patients on reduced dosage. Real-world data show that nintedanib seems to stabilize pulmonary function in progressive SSc-ILD, even at reduced doses, during 3 years of follow-up in about half of patients, without safety concerns when combined with biologic agents.

## Introduction

Systemic sclerosis is a multifaceted autoimmune connective tissue disorder characterized by microvascular dysfunction, immune dysregulation, and progressive fibrosis of the skin and internal organs and is associated with the highest mortality among rheumatic diseases [1, 2]. Among organ complications, interstitial lung disease (ILD) represents the principal determinant of morbidity and mortality, accounting for approximately one-third of disease-related deaths [3, 4].

Pulmonary abnormalities consistent with ILD are detected on high-resolution computed tomography (HRCT) in up to 70–80% of patients [5], however the clinical trajectory varies markedly. While some patients exhibit a relatively indolent or stable course, others develop a progressive fibrosing phenotype associated with functional decline, and increasing fibrotic lesions on HRCT [6, 7].

The pathogenesis of SSc-ILD involves immune activation, endothelial dysfunction, and excessive extracellular matrix deposition, driven in part by profibrotic pathways mediated through platelet-derived growth factor, fibroblast growth factor, and vascular endothelial growth factor signaling [8]. Nintedanib, an intracellular tyrosine kinase inhibitor targeting all these pathways, has demonstrated antifibrotic efficacy in idiopathic pulmonary fibrosis [9] and progressive fibrosing ILDs [10, 11]. In the SENSCIS trial, it significantly reduced the annual rate of forced vital capacity decline in SSc-ILD, including patients receiving concomitant mycophenolate [11].

Despite robust randomized trial evidence, data on long-term effectiveness, treatment persistence, and safety—especially in the context of combination with immunosuppressive regimens—are still limited. Observational data from real-world settings are therefore essential to better characterize treatment performance in broader and more clinically diverse patient populations.

The present study aimed to evaluate the long-term safety and effectiveness of nintedanib, as monotherapy or combined with immunosuppressive agents, in a real-world cohort of patients with SSc-ILD managed at a tertiary academic center.

## Methods

### Study design

Medical records of consecutive patients with systemic sclerosis, fulfilling the EULAR/ACR classification criteria [12], who received nintedanib for newly diagnosed fibrotic ILD or progressive ILD despite background immunosuppressive therapy, between January 2020 and December 2025 were reviewed. Progressive ILD was defined according to criteria of progressive fibrosing interstitial lung disease used in the INBUILD trial [10].

Demographics, clinical data and concomitant treatments were extracted from medical records. Pulmonary function tests, including forced vital capacity % of predicted (FVC%) and diffusing capacity for carbon monoxide % of predicted (DLCO%), were recorded 12 months prior to nintedanib initiation at baseline, and annually thereafter.

For the safety analysis, all patients who received at least one dose of nintedanib were included. Adverse events, dose adjustments, and treatment discontinuations were recorded. Severe diarrhea was defined according to CTCAE Grade 3 criteria as an increase of ≥ 7 stools per day over baseline and/or diarrhea requiring hospitalization or limiting self-care activities of daily living. A serious adverse event was defined as any untoward medical occurrence that at any dose resulted in death, was life-threatening, required inpatient hospitalization or prolongation of hospitalization.

For the efficacy analysis, we excluded those patients initiating immunosuppressive therapy simultaneously with nintedanib to reduce potential confounding effects and allow a more accurate assessment of the independent effect of nintedanib on lung function. Changes in pulmonary function tests from baseline (nintedanib initiation) were assessed at annual intervals thereafter. Lung function stabilization was defined as an FVC decline < 5% and a DLCO decline < 10% over the follow-up period [13].

### Statistical analysis

Continuous variables are presented as median (minimum–maximum). Normality of continuous variables was assessed using the Shapiro–Wilk test. Given the relatively small sample size and the non-normal distribution of several variables, comparisons of pulmonary function parameters across different time points were performed using the Wilcoxon signed-rank test, while comparisons between independent groups were performed using the Mann–Whitney U test. Categorical variables, including adverse event frequencies, are presented as counts and percentages and were compared using the chi-square or Fisher’s exact test, as appropriate. A two-sided p-value < 0.05 was considered statistically significant. Statistical analyses were performed using SPSS version 29.0.

### Ethical considerations

The study was conducted in accordance with the Declaration of Helsinki (October 2024 revision) and was approved by the Ethics Committee of Laikon University Hospital, National and Kapodistrian University of Athens (protocol number 267/26-04-2021). Informed consent was obtained from all participants for the use of their anonymized data.

## Results

### Patient Characteristics

Between January 2020 and December 2025, a total of 51 patients with systemic sclerosis (41 female, 10 male; 32 diffuse subtype) received nintedanib. The median age was 53 (range 18–78), and median duration was 6 (range 1–29) years. Patients were treated with nintedanib for a median of 33 (range 4–60) months.

Twenty-eight patients received nintedanib as an add-on to stable background immunosuppressive therapy, including mycophenolate mofetil (MMF) (*n* = 12), tocilizumab (*n* = 10), combination of MMF/tocilizumab (*n* = 3), rituximab (*n* = 1), and combination rituximab/MMF (*n* = 2). Eleven patients received nintedanib as monotherapy. The remaining 12 patients initiated nintedanib simultaneously with immunosuppressive therapy (MMF, *n* = 6; tocilizumab, *n* = 4; MMF plus tocilizumab, *n* = 2) and were excluded from the efficacy analysis. The dosage of mycophenolate mofetil was 2 g/day in all cases, while tocilizumab was administered at a dose of 162 mg subcutaneously once weekly. Rituximab was administered intravenously at a dose of 1 g on days 1 and 15 every 6 months. Dosing regimens remained overall stable throughout follow-up, except for 8 cases requiring temporary withholding of mycophenolate mofetil (*n* = 5) or tocilizumab (*n* = 3) due to respiratory infections. Regarding glucocorticoids, at nintedanib initiation, 22 patients were receiving prednisolone or equivalent at a median dose of 1.25 (range 0–10) mg/day. At last follow-up, 12 patients remained on the same dose, 5 had a dose reduction, and 5 had discontinued glucocorticoid treatment.

Demographics, disease characteristics and concomitant therapies are presented in detail in Table 1. No significant differences in disease characteristics or pulmonary function at nintedanib initiation were observed between the patients included in and excluded from the efficacy analysis (Table 1).

**Table 1:** Demographics, clinical characteristics and treatment regimens in 51 patients treated with Nintedanib for SSc-ILD

	All treatments	NTD monotherapy or NTD with stable background IMS	simultaneous onset of NTD and IMS	*p*
N	51	39	12	
Age (years), median (min, max)	53 (18, 78)	54 (26, 73)	49 (18, 78)	0.468
Disease duration (years), median (min, max)	6 (1, 29)	6.5 (1, 29)	5 (1, 19)	0.372
Sex (female), n (%)	42 (82)	32 (82)	10 (83)	0.786
Cutaneous subset Diffuse, n (%) Limited, n (%)	32 (63) 19 (27)	25 (66) 13 (54)	7 (34) 6 (46)	0.328 0.584
Anti-topoisomerase positivity, n (%) Anti-centromere positivity, n (%)	35 (68) 6 (12)	27 (71) 4 (12)	8 (61) 2 (22)	0.502 0.382
Pulmonary Hypertension, n (%)	5 (10)	4 (11)	1 (8)	0.622
Esophageal involvement, n (%)	34 (67)	23 (61)	11 (85)	0.103
Digital vasculopathy, n (%)	24 (47)	19 (50)	5 (40)	0.347
Diarrhea (prior to nintedanib), n (%)	7 (14)	5 (13)	2 (15)	0.581
Glucocorticoids, n (%) median dose (min, max), mg	22 1.25 (0,10)	17 2.5 (0,10)	5 1.25 (0,5)	0.441 0.694
Concomittant immunosuppression, n (%) Mycophenolate mofetyl, n (%) Tocilizumab, n (%) Rituximab, n (%) Mycophenolate mofetyl + Tocilizumab, n (%) Mycophenolate mofetyl + Rituximab, n (%)	40 (78) 18 (35) 14 (28) 1 (2) 5 (10) 2 (4)	28 (72) 11 (30) 10 (26) 1 (3) 3 (8) 2 (5)	12 (100) 7 (54) 4 (31) 0 (0) 2 (15) 0 (0)	0.048 0.177 0.734 1.000 0.591 1.000
FVC % at NTD onset, median (min, max)	73 (35, 105)	74.5 (35, 105)	72 (48, 94)	0.988
DLCO% at NTD onset, median (min, max)	43 (13, 78)	43 (13, 78)	38 (22, 58)	0.500

*DLCO* diffucing capacity for carbon monoxide, *FVC* forced vital capacity, *ILD* interstitial lung disease, *IMS* immunosuppression, *NTD* nintedanib, *SSc* Systemic Sclerosis

### Safety

Diarrhea was the most frequently reported adverse event, affecting 21 patients (41%), with severe diarrhea observed in 5 of them. Nausea and vomiting occurred in 5 (10%) patients. Nintedanib dosage was reduced in 19 patients (16 for diarrhea and 3 for nausea) to manage these adverse events and in most cases supportive treatment with antidiarreals or dietary adjustment was applied (Table 2). Laboratory abnormalities were uncommon; two patients developed elevated liver enzymes exceeding twice the upper limit of normal, without any other laboratory deviations. The majority of these adverse events occurred early after treatment initiation, typically within the first 3 months (Table 2).

**Table 2:** Adverse events recorded during follow-up with Nintedanib treatment

Adverse event		Time of occurrence after nintedanib initiation*	Dose reduction/ temporary withholding of nintedanib	Permanent discontinuation	Supportive treatment
Any diarrhea, n severe diarrhea, n	21 5	2.5 (1, 5) 2.0 (1, 3)	16/5 5/5	2 5	17 5
Nausea/vomiting, n	5	2.0 (1, 3)	3/1	1	1
Elevated liver enzymes, n	2	3.5 (3, 4)	0/0	0	0
Upper respiratory tract infections, n	8	10 (5, 24)	0/5	0	8

*values are expressed as median (min, max)

At last follow-up, 36 (70%) patients continued nintedanib, of whom 11 (22%) on a reduced dosage. Eight patients (16%) discontinued permanently therapy due to adverse events (7 due to diarrhea and 1 due to nausea) while the remaining 7 patients died during the study period due to cancer (*n* = 2), respiratory failure (*n* = 3), renal crisis (*n* = 1), and paralytic ileus (*n* = 1). Notably, no additional serious adverse events were observed among the 40 patients treated with combination regimens of nintedanib and immunosuppressive agents. During the follow-up period eight cases of uncomplicated upper respiratory tract infections, managed symptomatically or with antibiotics, were recorded.

### Efficacy

After excluding the 12 patients who started nintedanib simultaneously with immunosuppressive therapy and 4 patients who did not complete at least 1 year of nintedanib treatment due to adverse events (*n* = 3) or death (*n* = 1, renal crisis), 35 patients were included in the efficacy analysis. Among those, 15 and 20 patients completed 2 and 3 years of nintedanib treatment, respectively.

During the pre-treatment observation period (12 months prior to nintedanib initiation), median FVC% declined from 81 (range 37–115) to 75 (range 35–105) (*p* < 0.001, *n* = 35), and median DLCO% decreased from 53 (range 25–86) to 43 (range 18–78) (*p* < 0.001, *n* = 35). Following initiation of nintedanib, both FVC% and DLCO% remained stable after 24 months [median FVC% 78 (range 30–108); DLCO% 45 (range 12–29)]. In the subgroup of 20 patients completing 3 years of therapy, similar stabilization was observed; median FVC% changed from 75 (range 49–95) at baseline to 74.5 (range 32–96), and median DLCO% from 43 (range 25–62) to 45 (range 12,64) after 36 months respectively (Table 3). At follow-up end, lung function stabilized or improved in 23/35 patients, of whom 7 patients on nintedanib monotherapy, and deteriorated in 12/35.

A sub-analysis of patients receiving a reduced dosage of nintedanib who completed at least 2 (*n* = 16) or 3 (*n* = 7) years of treatment also demonstrated stabilization of lung function. Specifically, after 24 months, median FVC% changed from 71 (range 35–89) to 70 (range 26–98), and median DLCO% from 41.5 (range 18–78) to 38.5 (range 11–81). After 36 months, median FVC% changed from 70 (range 58–89) to 72 (range 44–88), and median DLCO% from 42 (range 25–48) to 34 (range 11–45), respectively. Accordingly, subgroup analysis by disease duration demonstrated stabilization in both patients with early (< 5 years) and longer-standing disease across all assessed time points. (Table 3).

**Table 3:** Changes in lung function tests at different timepoints in 35 patients with SSc-ILD treated with nintedanib and in subgroups according to nintedanib dosage and disease duration

Timepoint	-1 year	baseline	p*	1st year	p**	2nd year	p***	3rd year	p****
All patients (*n* = 35)									
FVC%	81 (37,115)	75 (35,105)	< 0.001	78 (30,108)	0.538	73 (25,104)	0.652		
FVC% (*n* = 20)		75 (49,95)						75 (32,96)	0.314
DLCO%	53 (25,86)	43 (18,78)	< 0.001	45 (12,99)	0.874	43 (11,84)	0.279		
DLCO% (*n* = 20)		43 (25,62)						45 (12,64)	0.197
Patients on maximum dosage (*n* = 19)									
FVC%	83 (50,115)	76 (39,105)	0.008	80 (30,108)	0.672	78 (25,104)	0.920		
FVC% (*n* = 13)		78 (49,95)						73 (32,96)	0.470
DLCO%	54 (40,78)	48 (23,67)	< 0.001	48 (20,99)	0.444	47 (16,84)	0.687		
DLCO% (*n* = 13)		46 (32,62)						47 (22,64)	0.658
Patients on reduced dosage (*n* = 16)									
FVC%	79 (37,76)	71 (35,89)	0.003	75 (32,92)	0.938	70 (26,98)	0.587		
FVC% (*n* = 7)		70 (58,89)						72 (44,88)	0.345
DLCO%	52.5 (25,86)	42 (18,78)	0.001	42 (12,67)	0.220	38 (11,81)	0.170		
DLCO% (*n* = 7)		42 (25,48)						34 (11,45)	0.104
Patients with early disease (*n* = 19)									
FVC%	84 (50,115)	74.5 (39,88)	< 0.001	78 (30,92)	0.348	77 (25,98)	0.616		
FVC% (*n* = 11)		72 (53,88)						71 (49,96)	0.721
DLCO%	50 (40,86)	43.5 (23,78)	< 0.001	45 (20,67)	0.517	44 (16,81)	0.448		
DLCO% (*n* = 11)		42.5 (32,52)						40 (22,64)	0.683
Patients with longer-standing disease (*n* = 16)									
FVC%	74 (37,109)	75 (35,105)	0.048	78 (32,108)	0.849	67 (26,104)	0.265		
FVC% (*n* = 9)		80 (49,95)						78 (32,93)	0.286
DLCO%	53 (25,76)	43 (18,67)	< 0.001	45 (12,99)	0.678	41 (11,84)	0.351		
DLCO% (*n* = 9)		50 (25,62)						45 (12,58)	0.083

All values are presented as median (min, max)

DLCO%: diffusing capacity for carbon monoxide % of predicted; FVC%: forced vital capacity % of predicted; SSc-ILD: systemic sclerosis-interstitial lung disease

* comparison between − 1 year and baseline

** comparison between baseline and 1st year

*** comparison between baseline and 2nd year

**** comparison between baseline and 3rd year

## Discussion

Evidence from three randomized clinical trials indicates that nintedanib preserves lung function in patients with progressive ILD with an acceptable safety profile [9–11]. However, their strict inclusion criteria and relatively short follow-up may affect generalizability to routine clinical practice. To date, real-world evidence on nintedanib in SSc-ILD remains limited to a small number of multicenter and registry-based observational studies most of which have relatively short follow-up periods [14–20]. To the best of our knowledge the present study is the first to provide long-term real world data demonstrating sustained effectiveness and high treatment presistence of nintedanib with an acceptable safety profile over extended follow-up in a heterogeneous, real-world SSc population.

In our study, gastrointestinal adverse events were the most common side effects with diarrhea and nausea/vomiting, affecting approximately 40% and 10% of patients. These results are in line with rates reported in the SENSCIS and INBUILD trial as well as in the aforementioned observational studies, in which gastrointestinal nintedanib-related adverse events ranged between 32% and 50% [14–20]. Mechanistically, diarrhea associated with nintedanib is likely multifactorial, reflecting inhibition of VEGFR-mediated signaling pathways involved in intestinal epithelial homeostasis, direct mucosal toxicity, and alterations in gut microbiota. However, the high prevalence of diarrhea after nintedanib treatment should also be interpreted in the context of the high baseline prevalence of gastrointestinal involvement in systemic sclerosis.

Importantly, the potential contribution of the nocebo effect [21] warrants consideration. A recent comparative analysis of placebo arms across different SSc trials demonstrated substantial variability in reported diarrhea rates, with the SENSCIS placebo arm exhibiting disproportionately high rates, almost four times higher [22]. The observed correlation between adverse event reporting and the framing or frequency of symptom descriptions in informed consent forms highlights the role of expectation-driven symptom reporting especially in a population with a high prevalence of gastrointestinal involvement. Taking all these into consideration, disentangling drug-related toxicity from disease-related manifestations and nocebo effects remains challenging and underscores the importance of careful patient counseling and standardized adverse event assessment.

Overall, treatment persistence in our cohort was notably high, with 70% of patients remaining on nintedanib after a median follow-up of 33 months. This finding compares favorably with the shorter-term real-world studies reporting 12-month nintedanib retention rates ranging from 75% to 88% and suggests that gastrointestinal adverse events, while frequent, are not treatment-limiting in most patients and may be effectively managed through symptomatic treatment or dose-adjustment strategies, likely reflecting an acceptable tolerability profile in light of the clinical benefit on lung function stabilization.

An important finding of our study is that long-term combination therapy with nintedanib and a single or double immunosuppressive regimen did not result in increased safety concerns, including infections or malignancies. A sub-analysis in the Senscis trial and some observational studies [14, 16, 23, 24] have reported on the safety of combining nintedanib mainly with mycophenolate but the duration of follow-up was limited, constraining the ability to assess possible long-term sequeale of combination treatments. Herein, we confirm that combination of nintedanib with immunosuppressives including biologic agents combined or not with MMF, is safe for at least 3 years. This finding is clinically important, as combination therapy is recommended for the management of rapidly progressive or refractory cases of SSc-ILD [25, 26] and is frequently required in daily practice for a prolonged period of time to inhibit both inflammatory and fibrotic pathways.

From an efficacy perspective, a sustained beneficial effect with stabilization of lung function in approximately 65% of patients up to three years is notable and compares favorably with data from clinical trials and real world observational studies. Importantly, the observation that dose reduction did not appear to compromise efficacy is particularly relevant for clinical practice, where tolerability often necessitates dose adjustments. This finding is consistent with post hoc analyses of SENSCIS [27] as well as with results from a real-world study including 51 patients with connective tissue disease-associated interstitial lung disease, 32 of whom had SSc [28], reinforcing the notion that maintaining treatment—albeit at a reduced dose—may still be clinically beneficial for these patients.

Another important finding is the apparent benefit in patients with long-standing disease. This contrasts with the population typically enrolled in RCTs, where applied criteria usually favor earlier disease stages. Our data suggest that antifibrotic therapy may retain efficacy even in more advanced fibrotic disease, supporting a broader therapeutic window than previously assumed. However, this interpretation should be made cautiously, as the absence of a control group limits the ability to distinguish treatment effects from the natural trajectory of disease stabilization in some patients.

Several limitations must be also acknowledged. The retrospective design introduces potential selection and information bias, while the relatively small sample size limits statistical power and subgroup analyses. The lack of a comparator group precludes definitive conclusions regarding treatment effect size, and heterogeneity in background therapies reflects real-world practice but complicates causal inference. In addition, the exclusion of patients initiating immunosuppressive therapy simultaneously with nintedanib may have influenced the composition of the efficacy cohort toward patients with less aggressive disease phenotypes, although no differences in pulmonary function at nintedanib initiation were observed between patients included in and excluded from the efficacy analysis. Furthermore, the progressive reduction in patient numbers throughout follow-up means that long-term analyses were more likely to include patients with better treatment tolerability and a more stable disease course. Taken together, the beneficial effect of nintedanib on pulmonary function observed over time should be interpreted cautiously, as its magnitude may have been overestimated and may not be fully generalizable to the broader population of patients with progressive SSc-ILD. Finally, the paucity of patients reported outcomes according to validated questionnaires like the St.John Respiratory Questionnaire and the analysis solely of FVC and DLCO, while standard, may not fully capture clinically meaningful changes in symptoms or quality of life of patients with SSc-ILD treated with Nintedanib.

In conclusion, our real-world data confirm the long-term safety profile of nintedanib in SSc-ILD, including cases receiving nintedanib in combination with biologic agents, as well as its beneficial effect on pulmonary function, with its benefits extending beyond the narrowly defined populations of clinical trials. These findings reinforce the role of antifibrotic therapy as a key component of the therapeutic armamentarium for progressive fibrosing ILD in systemic sclerosis. Prospective, multicenter studies incorporating standardized treatment protocols and patient-reported outcomes are needed to optimize therapy timing, combination strategies, and adverse event management.

## Data Availability

The data presented in the study are available on a reasonable request from the corresponding author.
